# The Level of Awareness Among Healthcare Practitioners Regarding the Relationship Between Breast Density and Breast Cancer

**DOI:** 10.7759/cureus.51282

**Published:** 2023-12-29

**Authors:** Renad F Althobaiti, Rehab Brnawe, Orjwan Sendi, Faikah Halawani, Alaa Marzogi

**Affiliations:** 1 College of Medicine and Surgery, Umm Al Qura University, Makkah, SAU; 2 Pharmacy, Umm Al Qura University, Makkah, SAU; 3 Physical Therapy, Umm Al Qura University, Makkah, SAU; 4 Radiology, Breast Imaging, King Abdullah Medical City, Makkah, SAU

**Keywords:** healthcare providers, general practitioners, awareness, breast density, breast cancer

## Abstract

Background

Breast cancer is the most prevalent cancer in women, accounting for around 23% of all cancer-related deaths across 140 nations. The awareness about breast density (BD) has a significant impact on early diagnosis of breast cancer.

Aim and objective

This study aims to assess the awareness of healthcare providers about BD in King Abdullah Medical City.

Methods

This is an analytical cross-sectional questionnaire-based study among the healthcare practitioners of KAMC in Makkah, Saudi Arabia. Questions measured knowledge about BD and a pass mark indicated participant awareness. The collected data were analyzed using SPSS, and a chi-square test used for bivariate analysis.

Results

Out of 124 participants, 41% were well aware. Physicians (37% of the sample) were significantly more aware than allied healthcare practitioners and nurses (awareness: 59.6%, 33.3%, 30.4% respectively, (p = 0.03)). Regarding specialty, radiologists and surgeons had the top level of awareness (62% and 64%, respectively) as compared to oncologists (47.1%) and other specialties (29.7%), (p= 0.016). Those above 40 years of age were more aware than those below 40 years (awareness: 62.1% and 34%, respectively, (p=0.007)). Non-significant factors included: gender, years of experience, screened versus non-screened, and receiving information before about BD (p > 0.05).

Conclusion

The results of this population-based study indicate the existence of moderate deficits in the general knowledge about BD and its relation to breast cancer. This might lead to a late diagnosis. The results showed no dramatic differences in the awareness among healthcare providers.

## Introduction

The most prevalent cancer in the world and the main reason for cancer mortality is breast cancer, accounting for around 23% of all cancer-related deaths across 140 nations. Its prevention is still difficult to achieve worldwide since it is a multi-step process involving several cell types. Early detection of breast cancer can increase relative survival rate [1].In the world as a whole, one in eight women are at risk of having breast cancer in their lifetime [2]. According to estimates, in Saudi Arabia, 17.3% of all new cancer cases (2282) in 2016 were breast cancer cases [3]. Early detection of malignant breast lesions helps reduce morbidity and mortality [2,4].

Two categories can be made up of all the risk variables that start the process. Age, sex, race, the genetic composition that encourages the familial occurrence of the neoplastic disease, and the occurrence of benign proliferative lesions of the mammary gland would all fall under the first category of intrinsic factors. They are all independent parameters that do not simply change a person's life. The second category would be extrinsic variables, which may have some degree of impact on the neoplastic process depending on lifestyles, food, or long-term medical interventions such as utilizing oral hormonal contraceptives or hormonal replacement treatment. Finding modifiable factors may aid in the creation of prevention methods that lower the incidence of breast cancer [5].

The most popular approach for screening breast cancer is standard (2D) mammography, which requires radiologists to inform patients about breast density (BD) and the potential need for additional screening [2]. This implies that BD may be a risk factor for breast cancer due to its ability to cover disease symptoms and act as a key risk factor for the development of cancer [5,6]. Dense breast is known to be one of the primary risks for the development of breast cancer [2]. Each woman has a different BD that can be only assessed under mammography. The level of BD can be affected by several factors including age, childbearing, menopausal status, hormone replacement therapy, and body mass index [2,7].

More than 90% of cancer fatalities are caused by women's neglect and ignorance, which frequently results in delayed diagnosis and advanced breast cancer presentations [1].

## Materials and methods

Data collection and population

Between July and August 2022, the present study attempted to assess healthcare practitioners' knowledge of BD and its relationship to breast cancer. The study included 124 practitioners and was approved by King Abdullah Medical City in Makkah, Saudi Arabia (Approval number: 22-968).

An analytical cross-sectional observational design was employed. Because the study was conducted at a single center, King Abdullah Medical City, in Makkah over a brief period of time, it was able to estimate the level of knowledge among medical professionals regarding BD as a risk factor for breast cancer.

All healthcare practitioners in King Abdullah Medical City were included, with the only exclusion being the refusal to participate in the study (n=2). As previously stated, the sample size was 124, and it was a probability sampling that was measured by n = N / (1 + N e^2^) equation. The majority of respondents were females (78.4%) followed by males (21.6%), we also observed that females were more enthusiastic than males to participate. This demonstration was seen because breast cancer is more likely to occur in females than males. The practitioners' educational level in the health field was at least a diploma degree.

The data for this study was collected in a survey of two sections. The first section included sociodemographic questions such as gender, age, job field, department of work in a hospital, and years of clinical practice. Another section included to assess the main objective containing nine questions written by the author, such as whether they had a breast examination before by mammogram, or received any information about BD or mammogram before, where they got the information, and whether the dense breast will require more examination for early diagnosis. We also considered their perspectives on the effect of hormone replacement therapy in dense breasts, breast cancer diagnosis in dense breasts, and image clarity of dense breasts in mammograms. Furthermore, the associations between dense breasts and the risk of breast cancer, age, childbearing, and breast size.

Google Forms were used to collect and maintain the data. Informed consent was included on the cover page in two languages Arabic and English, and the data collectors used a barcode connected directly to Google Forms to reduce the number of refusal practitioners because the majority of practitioners were at work, though the barcode helped to delay practicing until their breaktime and prevented any distraction to them. The data were collected from a random sample to estimate the prevalence of unknown parameters from the target population.

Data analysis

Statistical analysis was performed using IBM SPSS version 25 computer statistical software package, frequency test for univariate analysis, and chi-square test for bivariate analysis. A p-value of < 0.05 was considered statistically significant. The study reported a confidence interval of 95%.

## Results

Although BD information has been mandated to be disclosed worldwide, it remains largely unknown whether BD can affect breast cancer detection and what impact it has on risk. After getting the informed consent of individuals, 124 people completed the questionnaire. At the start of the study, all completed questionnaires were included, and any partially completed questionnaires were excluded. With the aid of SPSS software, the amassed data were processed and analyzed.

Participants

Descriptive statistics were performed to describe the sociodemographic data of the individuals. Statistical significance was defined as p< 0.05, and factors connected to the outcome measures were identified using a 95% confidence interval (CI). Of the 124 participants, 92 (74.2%) were women, while 32 (24.8%) were men. As for age, 95 (76.7%) were <40 years while 29 (23.4%) were >40 years. Among the healthcare workers, physicians were 47 (37.9%), nurses were 24 (19.4%), and allied healthcare professionals constituted 24 (19.4%), these were the most commonly stated job categories. Administration accounted for eight (6.5%), pharmacy seven (5.6%), and other occupations for 14 (11.3%). The participants' departments were Radiology department 21 (16.9%), Oncology 17 (13.7%), Surgery 11 (8.9%), and 75 (60.5%) in other departments. Eighty (64.5%) of the participants had <10 years in clinical practice, while 44 (35.5%) had >10 years (Table 1).

**Table 1:** Sample demographics

	N(%)
Gender	
Female	92 (74.2%)
Male	32 (25.8%)
Age	
Below 40 years	95 (76.7%)
Above 40 years	29 (23.4%)
Job Field	
Physicians	47 (37.9%)
Allied healthcare professionals	24 (19.4%)
Nurses	24 (19.4%)
Administrations	8 (6.5%)
Pharmacy	7 (5.6%)
Others	14 (11.3%)
Department	
Radiology	21 (16.9%)
Oncology	17 (13.7%)
Surgery	11 (8.9%)
Others	75 (60.5%)
Years in Clinical Practice	
Less than 10 years	80 (64.5%)
More than 10 years	44 (35.5%)

BD awareness

This study showed that 43 (34.7%) of the participants knew that hormone replacement therapy would increase BD while eight (65.3%) did not. Concerning the relation between BD and breast cancer diagnosis, 52 (41.9%) knew breast cancer diagnosis would be harder in breasts with high density, while 72 (58.1%) were not aware of that. Seventy-two (58.1%) knew high BD will require more examination for early diagnosis, while 52 (41.9%) did not. Forty respondents (32.3%) knew the image of the breast (mammogram) would look less diagnostic in high-density breasts, while 84 (67.7%) did not. Fifty-five (44.4%) knew that the greater BD, the greater the risk of breast cancer, while 69 (55.6%) were unaware. Forty-one (33.1%) knew the relation between age and BD, which decreases with aging, while 83 (66.9%) were unaware. About the correlation between BD and childbearing, 39 (31.5 %) knew the relation between childbearing and BD, which increases with childbearing, while 85 (68.5%) were unaware. Thirty-five (28.2%) knew no relation between BD and breast size, while 89 (71.8%) were unaware. Twenty-five (20.2%) recommended conducting a breast cancer risk assessment as the first supplemental screening, while 99 (79.8%) were unaware (Table 2).

**Table 2:** Knowledge Regarding Breast Density among Study Participants

Questions	True/False	Values
What is the impact of hormone replacement therapy on breast density?	True	43 (34.7%)
	False	81 (65.3%)
Do you think that there may be a diagnosis of breast cancer?	True	52 (41.9%)
	False	72 (58.1%)
In your opinion if the breast density is high will it require further investigations?	True	72 (58.1%)
	False	52 (41.9%)
In your opinion how the image of breast (mammogram) will look like?	True	40 (32.3%)
	False	84 (67.7%)
In your opinion what is the relation between the risk of breast cancer and breast density?	True	55 (44.4%)
	False	69 (55.6%)
In your opinion what is the relation between breast density and age?	True	41 (33.1%)
	False	83 (66.9%)
In your opinion what is the relation between breast density and childbearing?	True	39 (31.5%)
	False	85 (68.5%)
In your opinion what is the relation between breast density and breast size?	True	35 (28.2%)
	False	89 (71.8%)
Amirah is a 60-year-old patient of yours who comes to discuss supplemental screening with you. She is confused about what this means. What would you recommend for Amirah?	True	25 (20.2%)
	False	99 (79.8%)

BD knowledge

A comparative study of the relationship between knowledge and participant demographics shows moderate knowledge gaps regarding BD and its connection to breast cancer. Physicians were significantly more aware (n=28, 59.6%) than allied healthcare practitioners (n=8, 33.3%), and nurses (n=7,30.4%). In administration, only one was aware (12.5%), in pharmacy only two (28.6%), and in other professions two (28.6%) with p = 0.03, meaning, it is significantly associated. Regarding specialty, Radiologists and Surgeons had the top level of awareness (62% and 64%, respectively) as compared to oncologists (47.1%) and other specialties (29.7%), with p= 0.016, thus a significant association. Those >40 years of age were more aware than those <40 years (awareness: 62.1% and 34%, respectively) with p=0.007, thus no significant association. Non-significant factors included gender, years of experience, screened versus non-screened, and receiving information about BD, with p > 0.05, thus no significant association (Table 3).

**Table 3:** Distribution of Participants' Knowledge Regarding Breast Density by Their Characteristics

	Aware	Unaware	p-value
Age			
Below 40	32 (34%)	62 (66%)	p-value=0.007
Above 40	18 (62.1%)	11 (37.9%)	
Gender			
Female	37 (40.7%)	54 (59.3%)	P-value=0.997
Male	13 (40.6%)	19 (59.4%)	
Job Field			
Physician	28 (59.6%)	19 (40.4%)	P-value=0.030
Allied healthcare professionals	8 (33.3%)	16 (66.7%)	
Nurse	7 (30.4%)	16 (69.6%)	
Administration	1 (12.5%)	7 (87.5%)	
Pharmacy	2 (28.6%)	5 (71.4%)	
Others	4 (28.6%)	10 (71.4%)	
Years in Clinical Practice			
Less than 10 years	27 (34.2%)	52 (65.8%)	P-value=0.050
More than 10 years	23 (52.3%)	21 (47.7%)	
Department			
Radiology	13 (61.9%)	8 (38.1%)	P-value=0.016
Oncology	8 (47.1%)	9 (52.9%)	
Surgery	7 (63.6%)	4 (36.4%)	
Others	22 (29.7%)	52 (77.3%)	
Screening by Mammogram			
Screened	11 (45.8%)	13 (54.2%)	P-value=0.548
Non-screened	26 (38.8%)	41 (61.2%)	
Received Previous Information			
Yes	36 (47.4%)	40 (52.6%)	P-value=0.054
No	14 (29.8%)	33 (70.2%)	
Sources of Received Previous Information			
Educational sources	19 (48.7%)	20 (51.3%)	P-value=0.986
Physician- health practitioner	12 (48%)	13 (52%)	
Social media	3 (50%)	3 (50%)	
Relatives- Friends	2 (40%)	39 (52%)	

The majority of participants, according to the demographics, were unaware of the benefits, the ideal timing, or the correct techniques for measuring BD. The general population and those in the health sector should get health information on risk elements associated with breast cancer and methods for early diagnosis from qualified healthcare experts.

## Discussion

BD is defined as the ratio of radiolucent fatty elements to radiopaque both epithelial and stromal tissue elements as seen on the mammogram [8,9]. The Breast Imaging and Data Reporting System (BI-RADS) Atlas classifies BD as (a) almost fatty, (b) fibroglandular tissue scattered, (c) heterogeneous that might conceal minor masses, and (d) extremely dense (ACR BI-RADS) [10]. In this study, the awareness and knowledge of healthcare providers (HCPs) regarding BD and its association with breast cancer were investigated. Findings showed that the majority of the HCPs were not aware that with high BD, diagnosis of breast cancer becomes difficult (n=72, 58.1%) and the image of the mammogram will be less clear (n=84,67.7%). A similar study was conducted by Brown and colleagues wherein the majority of the physicians were not aware of the risk of BC because of dense breasts. Furthermore, their study found that primary care providers (PCPs) were more unaware of BD laws than the specialists [11,12]. Several studies proved or concluded that high BD could make breast cancer less sensitive to detection thus increasing the risk of missed cancer [12,13]. Furthermore, researchers identified BD as a distinct breast cancer risk factor [14-16].

Factors such as age, childbearing, and breast size in relation to BD were also asked of participants. The study revealed that most of the participants were not aware that BD decreases with age, increases with childbearing, and also has no relation with breast size. Studies done in different regions observed a significant association of BD with age in which BD decreases as women age especially those in the menopausal stage [17-19]. In relation to childbearing, the study of Yaghjyan et al. found a positive relation between childbearing in terms of breastfeeding and BD of premenopausal women [20].

Knowledge about BD was assessed in the current study by comparing the knowledge of HCPs based on demographic information. Findings showed that physicians were significantly more aware among other participants while surgeons were more aware compared to radiologists, oncologists, and other specialties. It was also observed that participants aged above 40 years old were more aware than those below 40 years old. In a qualitative interview study conducted in Australia, general practitioners had limited knowledge of BD and little experience talking about BD to women. The findings in the current study also indicate HCPs’ limited awareness and knowledge about BD. In contrast to our study, a cross-sectional study of PCPs in the USA showed a high percentage of Australian GPs knowledge and positive attitude in informing their patients about BD [21]. The varying results may be due to formal training or guidelines provided to HCPs about BD or the mandated BD notification in the USA.

There has been an increase in global discussion of BD, which includes public dissemination about BD and post-legislation of BD notification in the United States. In other countries, BD notification does not occur resulting in varying views, understanding, and knowledge of women and HCPs about BD and its association with breast cancer. The current study has some potential limitations. First, response bias is possible in this study since this is a self-reporting survey. Second, the participants were from a single center thus results may not reflect the general knowledge and awareness of the population in Saudi Arabia. The HCPs in this study majority are female and have less than 10 years of clinical practice thus may not be representative of all Saudi Arabian HCPs. Nevertheless, we think that the study survey is thorough in terms of respondents and settings, enabling us to evaluate practitioners' knowledge.

## Conclusions

The results of this population-based study indicate the existence of moderate deficits in the general knowledge about BD and its relation to breast cancer. This might lead to a late diagnosis. The results showed no dramatic differences in awareness among HCPs.
